# Transcriptomic responses of the calanoid copepod *Calanus finmarchicus* to the saxitoxin producing dinoflagellate *Alexandrium fundyense*

**DOI:** 10.1038/srep25708

**Published:** 2016-05-16

**Authors:** Vittoria Roncalli, Matthew C. Cieslak, Petra H. Lenz

**Affiliations:** 1 Békésy Laboratory of Neurobiology, Pacific Biosciences Research Center, School of Ocean and Earth Science and Technology, University of Hawaii at Manoa, Honolulu, HI 96822, USA

## Abstract

In the Gulf of Maine, the copepod *Calanus finmarchicus* co-occurs with the neurotoxin-producing dinoflagellate, *Alexandrium fundyense*. The copepod is resistant to this toxic alga, but little is known about other effects. Gene expression profiles were used to investigate the physiological response of females feeding for two and five days on a control diet or a diet containing either a low or a high dose of *A. fundyense*. The physiological responses to the two experimental diets were similar, but changed between the time points. At 5-days the response was characterized by down-regulated genes involved in energy metabolism. Detoxification was not a major component of the response. Instead, genes involved in digestion were consistently regulated, suggesting that food assimilation may have been affected. Thus, predicted increases in the frequency of blooms of *A. fundyense* could affect *C. finmarchicus* populations by changing the individuals’ energy budget and reducing their ability to build lipid reserves.

Major shifts in species distributions in pelagic ecosystems have been attributed to complex changes in the abiotic and biotic environment in response to global climate change^1^. Although such biogeographic shifts in pelagic communities have been widely documented, they cannot be explained by the species’ physiological tolerances of physical and chemical factors, such as temperature and salinity^1^. Thus, a species may change its biogeographic range not because environmental factors are outside of its physiological tolerance, but because conditions are sub-optimal, and it cannot maintain its competitive advantage. The physiological compromises that occur when conditions are sub-optimal are poorly understood, particularly for marine zooplankton species, such as copepods^1^,^2^.

One reported change in marine environments is a worldwide increase in the range and number of harmful algal blooms^3^ and this trend is expected to continue^4^. These toxic primary producers are ingested by herbivorous copepods, which accumulate the toxins and contribute to their transfer to higher trophic levels, often with catastrophic effects, i.e., increased mortality in fishes, marine mammals, birds and even humans^4^,^5^,^6^,^7^. During the summer in the Gulf of Maine, the calanoid copepod *Calanus finmarchicus* co-occurs with the toxic dinoflagellate *Alexandrium fundyense*, which is responsible for outbreaks of paralytic shellfish poisoning^7^,^8^,^9^,^10^. Although the dinoflagellate has no effect on *C. finmarchicus* survival^11^, it decreases the copepod’s fitness by reducing its reproduction success after multi-day exposure^12^; however, little is known about how the dinoflagellate affects the physiology of the copepod.

In the last decade, high-throughput sequencing has greatly expanded our ability to investigate organism-environment interactions by measuring patterns in gene expression^13^. Gene expression analyses can now be studied in ecologically important non-model species such as *C. finmarchicus*, in order to determine how they adjust physiologically to natural fluctuations in their surroundings, and identify factors that constitute “environmental stressors”^13^,^14^. The typical physiological response to a stressor is the “cellular stress response” (CSR), which involves changes in the expression of 1,000 or more genes^13^,^15^,^16^,^17^. Features of the CSR are: 1) up-regulation of stress proteins to counteract damage to cellular macromolecules; 2) redistribution of metabolic resources away from ‘energetically expensive’ functions; 3) the arrest of the cell cycle; and 4) apoptosis (in the case of extreme conditions)^13^.

The goal of this study was to identify physiological pathways that were regulated in *C. finmarchicus* in response to the introduction of *A. fundyense* into the diet. RNA-Seq was used to determine relative gene expression in adult females after 2 and 5 days feeding on either a control or one of two experimental diets. Functional analysis was used to test the hypotheses that the copepod physiological response would be characterized by 1) the up-regulation of detoxification pathways as part of a general cellular stress response (CSR) and 2) the persistence of up-regulation of detoxification pathways over time

## Results

*Calanus finmarchicus* adult females were fed for either 2- or 5-days on a control, low dose or high dose diet of *Alexandrium fundyense* (Table 1). Survival was high in all experimental jars with mortalities of 0% at 2 days and <9% at 5 days. At the end of the two incubations, females from each treatment were pooled for high-throughput RNA-Seq.

### Sequencing, mapping and differential gene expression

Illumina sequencing generated over 484 million paired-end 100 bp reads (18 libraries = 3 replicates × 3 treatments × 2 time points) with 15 to 36 million reads per library and an average of 26 million across all samples (Supplementary Table 1). For each sample, 10 to 24 million quality-filtered reads were mapped against the *C. finmarchicus* reference transcriptome (96,090 contigs)^18^. Mapping resulted in an overall alignment rate of 71 to 72% per sample, with fewer than 1% of the reads mapped more than once (Supplementary Table 1). The depth of mapping (>10 millions of mapped reads) here has been estimated to be sufficient for quantitative gene expression of 80% of the genes in other eukaryotes^19^,^20^. Using the expression cut-off of 1 cpm (count per million), statistical testing of differentially expressed genes (DEGs) was performed on a total of 28,756 for the 2-day and 27,943 genes for the 5-day datasets. The remaining genes in the reference transcriptome were either not expressed in adult females (ca. 1/3 of transcripts)^18^, or expression was below the filter cutoff (1 cpm).

### Differential gene expression as a function of time

**Figure 1:**
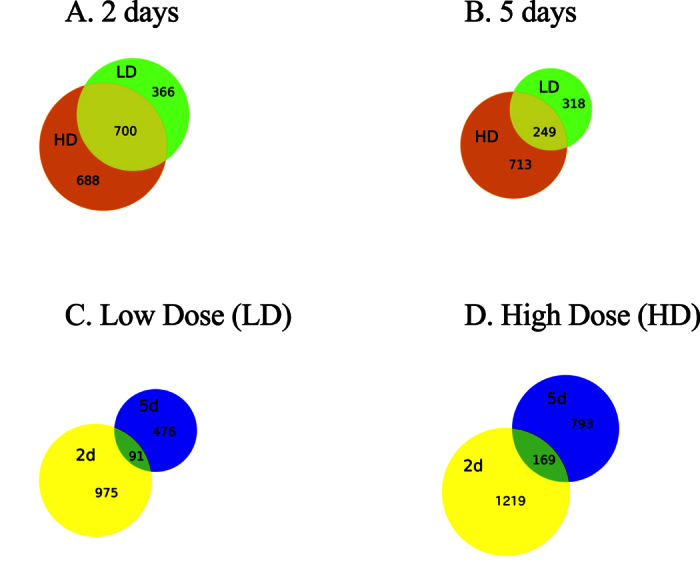
Venn diagrams of differentially expressed genes (DEGs) in *C. finmarchicus* adult females feeding on *A. fundyense* diets (LD, HD) for either 2 or 5 days. (**A**) Comparison between LD and HD diets at 2 days. (**B**) Comparison between LD and HD diets at 5 days. (**C**) Comparison between 2 and 5-day time points for LD treatment. (**D**) Comparison between 2 and 5-day time points for HD treatment.

The number of DEGs in the control vs. LD and control vs. HD were higher at 2 days than at 5 days (Fig. 1, Table 2). At 2 days the number of DEGs represented 4 to 5% of the transcripts with mapped reads while at 5 days this decreased to 2 to 3% (Table 2). Over 50% of the DEGs were shared between the two treatments at 2 days. At 5 days, this decreased to 26% and 44% for the control vs. HD and control vs. LD comparisons (Fig. 1). The number of shared DEGs between time points was low, suggesting that the physiological response changed between 2 and 5 days (Fig. 1). Statistical comparison between LD and HD females indicated many fewer DEGs between experimental treatments at 2 days, while at 5 days the number of DEGs in the C vs. LD and LD vs. HD comparisons were similar (Table 2).

**Figure 2:**
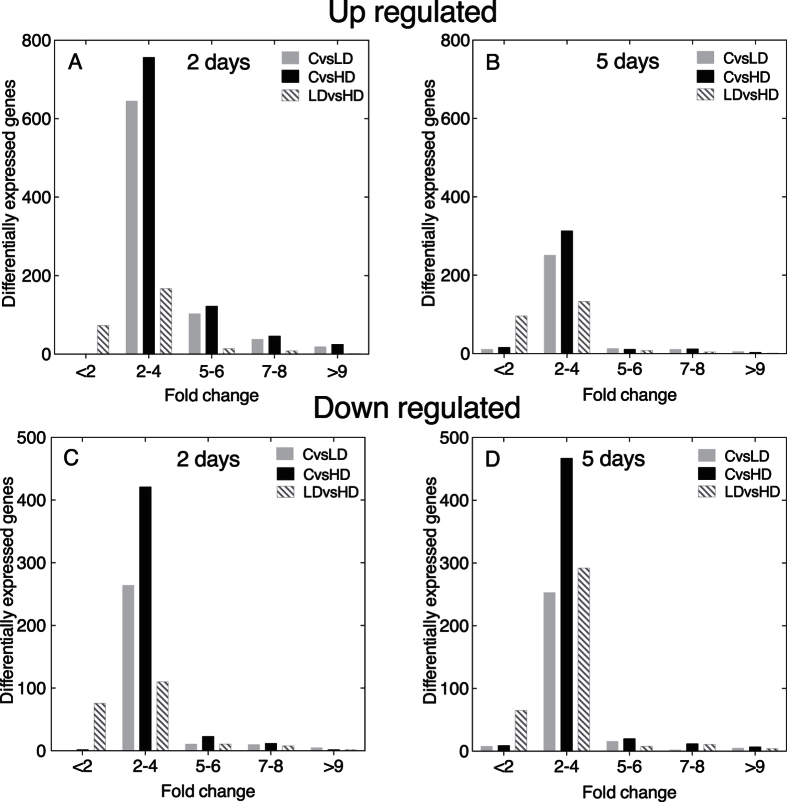
Magnitude of response of differentially expressed genes (DEGs) in *C. finmarchicus* adult females feeding on *A. fundyense* (LD, HD) for either 2 or 5 days. Differentially expressed genes were identified by comparing control vs. LD (CvsLD), control vs. HD (CvsHD) and LD vs. HD. (**A**) Fold-change difference in expression for up-regulated DEGs at 2 days. (**B**) Fold-change difference in expression for up-regulated DEGs at 5 days. (**C**) Fold-change difference in expression for down-regulated DEGs at 2 days. (**D**) Fold-change difference in expression for down-regulated DEGs at 5 days.

The direction and magnitude of the response is shown in Fig. 2. The magnitude of the response was modest with differential gene expression between the experimental and control diets less than 4-fold different for the majority of up- and down-regulated genes, as well as for both treatments and time points (Fig. 2). The number of up-regulated genes was significantly lower at 5 days than at 2 days for all paired comparisons (*X*^2^ test; control vs. LD: p < 0.0001, control vs. HD: p < 0.0001,and LD vs. HD: p ≤ 0.031). The number of down-regulated genes was significantly lower at 5 days in the control vs. LD comparison (*X*^2^ test, p < 0.01), but not in the control vs. HD (*X*^2^ test, p = 0.194). For the LD vs. HD comparison, the number of down-regulated DEGs was greater at 5 days than at 2 days, and this difference was significant (*X*^2^ test, p < 0.0001), suggesting a dose dependent response. The pattern of a large number of DEGs (>1,000) at 2 days, and fewer DEGs and a more pronounced dose-response at 5 days are consistent with an initial cellular stress response, followed by a cellular homeostatic response.

### Functional annotation of differentially expressed genes (DEGs)

**Figure 3:**
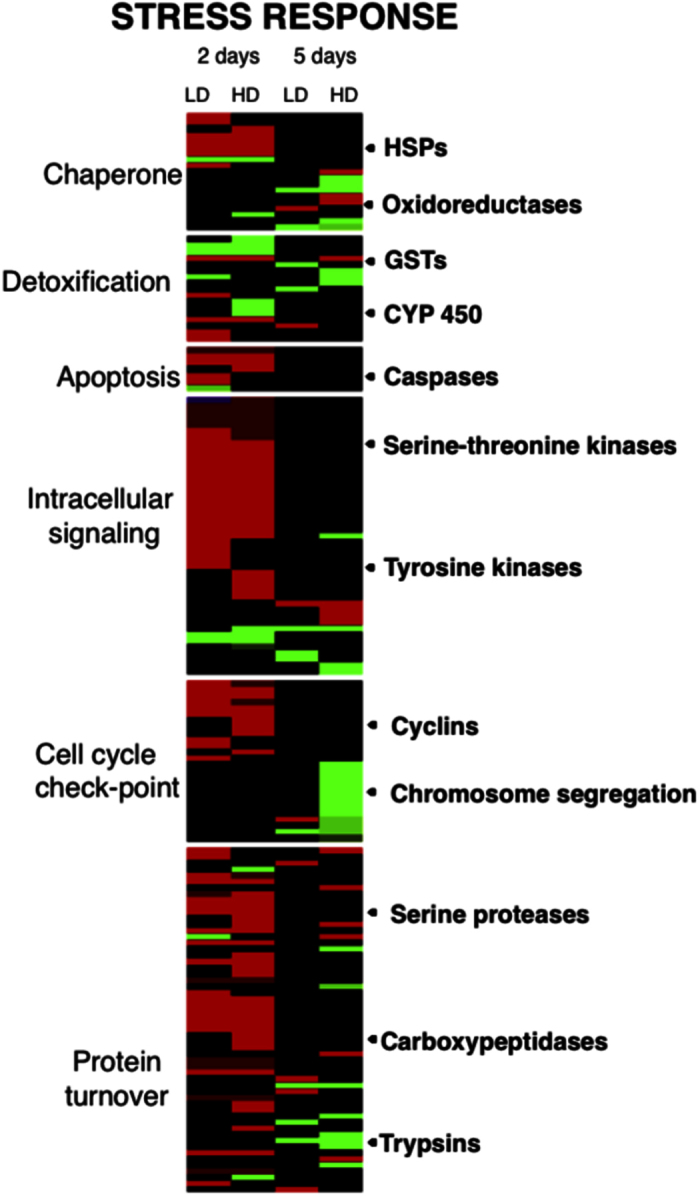
Differential expression of stress related genes. Heat map for DEGs involved in cellular stress response of *C. finmarchicus* adult females feeding on *A. fundyense* diets (LD, HD) for either 2 or 5 days. Heat maps were generated using the *heatmap.2* function implemented in R software. Columns are ordered by time points (2 and 5 days) and by treatment (LD and HD) as labeled. Genes were clustered by functional annotation in the groups: chaperone, detoxification, apoptosis, intracellular signaling, cell cycle check-point and protein turnover (labels on the left). Color-coding for relative expression rate (absolute fold change) between experimental treatment and control is shown on the bottom right.

**Figure 4:**
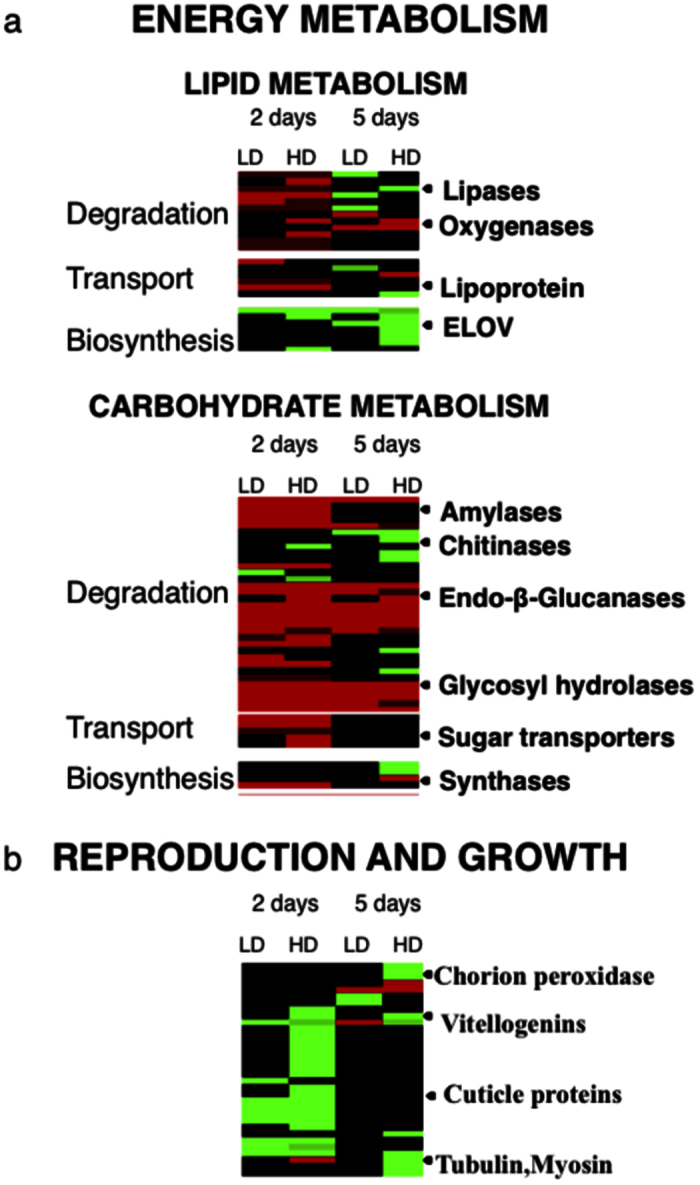
Differential expression for energy metabolism and growth related genes. (**A**) Heat map for DEGs in *C. finmarchicus* adult females feeding on *A. fundyense* diets (LD, HD) for either 2 or 5 days involved in energy metabolism that included lipid and carbohydrate metabolisms; genes were clustered by functional annotation in the groups: degradation, transport and biosynthesis (labels on the left). (**B**) Heat map for DEGs in *C. finmarchicus* adult females feeding on *A. fundyense* diets (LD, HD) for either 2 or 5 days involved in reproduction and growth. Heat maps were generated using the *heatmap.2* function implemented in R software. Columns are ordered by time points (2 and 5 days) and by treatment (LD and HD) as labeled. Relative expression rate (absolute fold change) is calculated for females feeding on the toxic diet compared to adult females.

In order to investigate the biological processes that were regulated in *Calanus finmarchicus* females in response to the toxic dinoflagellate on shorter (2 days) and longer (5 days) time scales, the DEGs with significant annotations were organized according to their biological function. Blast annotation was retrieved for a total of 1,162 DEGs (42%). The DEGs represented a broad range of conserved eukaryotic processes, including many involved in intracellular signaling, protein turnover, energy metabolism, reproduction and growth (Figs 3 and 4). The DEG pattern presented here supports the interpretation that the females’ initial response is comparable to the “cellular stress response” (CSR), which was followed by a physiological adjustment known as the “cellular homeostatic response” (CHR) at 5 days. The two time points were characterized by differences in DEGs related to metabolic activity (lipids, carbohydrates), which was confirmed by enrichment analysis (Tables 3 and 4).

### Evidence for cellular stress response (CSR) at 2 days

Upon initial exposure to *Alexandrium fundyense* diets (LD, HD), 117 genes, which are typical of the cellular stress response, were differentially regulated in *C. finmarchicus* adult females compared with the control (Fig. 3). These included DEGs involved in processes such as molecular chaperoning, apoptosis, cell cycle checkpoint, intracellular signaling and protein turnover (Fig. 3). A predominant signature of this initial response was that up-regulated DEGs included several heat shock proteins (HSP40, 70), serine and tyrosine kinases, proteases and carboxypeptidases (protein turnover); many of these genes were shared between the two toxic treatments (Fig. 3). Enrichment analysis confirmed that many processes associated with protein turnover such as “RNA biosynthetic process”, “cellular protein metabolic process”, “translation” and “macromolecule complex” were significantly up-regulated in both treatments compared with the control, but not in the LD vs. HD comparison (Table 3).

Consistent with an increased energy requirement associated with the CSR, regulation of DEGs involved in energy metabolism was observed in females on either one of the two experimental treatments. In both lipid and carbohydrate metabolism, genes involved in degradation were up-regulated, while genes involved in biosynthesis were down-regulated (Fig. 4). Lipid metabolism played a key role in the copepod response to *A. fundyense* and this process was enriched among the down-regulated genes in the HD treatment (Table 3). DEGs involved in lipid degradation included several lipases, phospholipases, acyltransferases and lipoxygenases, which showed similar expression patterns in the 2 treatments in terms of number of DEGs and relative fold-change (Fig. 4). Similar to lipid metabolism, carbohydrate degradation was also represented in this initial response (Fig. 4). Of the total number of DEGs in carbohydrate metabolism, 65% were involved in degradation and digestion that included several cellulases, amylases, endo β glucanases and glycosyl hydrolases (Fig. 4). Interestingly, several of these DEGs were shared among the four experimental conditions (see below). In addition, we observed down-regulation of transcripts associated with reproduction and growth such as multiple vitellogenins and cuticle proteins (Fig. 4). This response was most pronounced in the HD treatment, and several of these genes were differentially expressed between the LD and HD treatments.

### Evidence of cellular homeostatic response (CHR) at 5 days

At 5 days, many fewer DEGs (57) were involved in processes that characterize the stress response (e.g. molecular chaperoning, protein turnover), and many of these were now down-regulated (Fig. 3). Enrichment analysis confirmed this switch from up- to down-regulated DEGs in these processes between the 2-day and 5-day time points in both LD and HD treatments (Fig. 3, Tables 3 and 4). This was particularly evident in the HD treatment where “translation”, “RNA biosynthetic process” and “lipid metabolic process” had changed from up-regulated to down-regulated (Tables 3 and 4). Other down-regulated genes at 5 days included those involved in cell cycle check-point (chromosome segregation proteins) and intracellular signaling (tyrosine kinases) (Fig. 3). These were specific to the HD treatment, and the majority was also among the DEGs in the LD vs. HD comparison. In addition, down-regulation was observed for some digestive enzymes, e.g., trypsins (3/7 DEGs; Fig. 3). The exception to this pattern was the presence of several up-regulated serine kinase transcripts, which are involved in intracellular signaling (Fig. 3).

Lipid metabolism continued to play a significant role in the copepod physiological response in both treatments as indicated by the enrichment analysis (Table 4). Among lipid degradation transcripts, there were fewer up-regulated genes, and an overall increase in the down-regulated ones, including several that had switched from up- to down-regulated (Fig. 4). The number of up-regulated genes decreased to just 1 and 4 in the LD and HD treatments, respectively (Fig. 4).

Down-regulation of lipid biosynthesis was more pronounced at 5 days: a larger number of DEGs (16 DEGs compared with 6 DEGs at 2 days), higher fold-change (5–6 fold change difference) and a pronounced dose effect (Fig. 4). Of the 16 DEGs involved in lipid biosynthesis, 11 were only regulated at this time point (5 days), with down-regulation of transcripts like ELOV 4, ELOV 6 and steroid dehydrogenase being dose dependent (Fig. 4). Similarly, the number of up-regulated DEGs involved in carbohydrate metabolism decreased between time points, while there was an increase in the down-regulated ones, which included several chitinases (Fig. 4). Four of the five were differentially expressed in the HD treatment only (Fig. 4). Down-regulation was also observed in carbohydrate biosynthesis again with several synthases only regulated in the HD treatment (Fig. 4).

Down regulation of genes involved in energetically expensive processes such as reproduction and growth persisted at the 5-day time point with vitellogenins, cuticle proteins, tubulins and myosins down-regulated specifically in the HD treatment, three genes (two vitellogenins and one cuticle protein) were also among the DEGs in the LD vs. HD comparison (Fig. 4). Enrichment analysis confirmed that reproduction was significantly represented among the down-regulated genes in the HD treatment (Table 4).

### Detoxification

**Figure 5:**
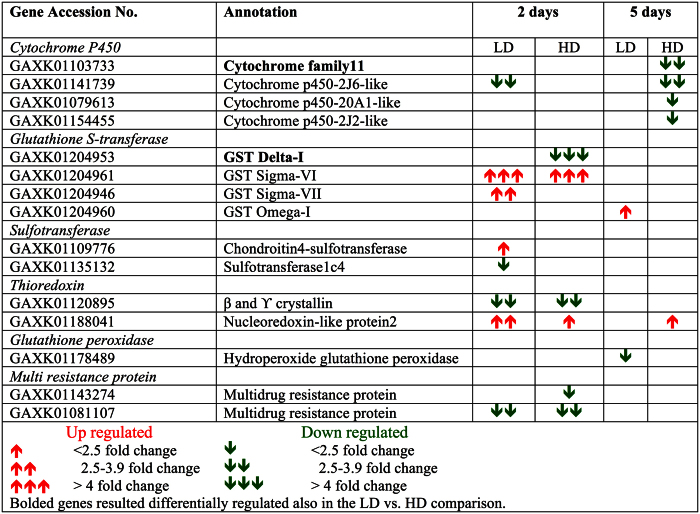
List of detoxification enzymes differentially expressed in *C. finmarchicus* feeding on *A. fundyense* diets 15 putative transcripts involved in detoxification (phase I, II, III) that resulted differentially regulated in females feeding on *A. fundyense* (LD, HD) for either 2 or 5 days compared with adult females feeding on the control diet. For each gene, Accession No. (NCBI), *blastx* annotation and relative fold change in expression are listed. The direction of expression (up- down-regulated) and the magnitude are indicated by arrows red = up- and green = down-regulated genes).

The *Calanus finmarchicus* reference transcriptome used in this study included more then 200 of genes involved in detoxification such as superoxide dismutases (SOD), ferritins, catalases, cytochromes P450, aldehyde dehydrogenases and 39 glutathione S-transferase^18^,^21^. Surprisingly, detoxification was not a major component of the *C. finmarchicus* response to the toxic dinoflagellate: only a small number of DEGs were involved in detoxification with the majority of them being down-regulated (Fig. 3) and detoxification was absent from the list of “enriched” GO terms. Similarly, the “parent” biological process “response to stimulus” was not enriched at either the 2- or 5-day time points (Tables 3 and 4). The DEGs included four members of the cytochrome P450 family, four glutathione S-transferases (GST), glutathione peroxidase, sulfotransferase and thioredoxin (phase II) and multi-resistance proteins (MXR) (phase III) (Fig. 5). Detoxification is commonly associated with the CSR, and the response at the 2-day time point (Fig. 5) could have been part of the general stress response, since the DEGs changed between time points with the majority of genes (8/15 DEGs) regulated exclusively after the initial exposure and only 2 DEGs (CYP450 and nucleoredoxin) expressed at both time points (Fig. 5). Although some of the genes were up-regulated (2 days), down-regulation was predominant at both time points with a dose response observed at 5 days (Fig. 5). The response of the majority of these genes was similar in the LD and HD treatments, as suggested by their absence from the list of DEGs in the LD vs. HD comparisons at the two time points (Fig. 5).

### DEGs shared across all experimental conditions

**Figure 6:**
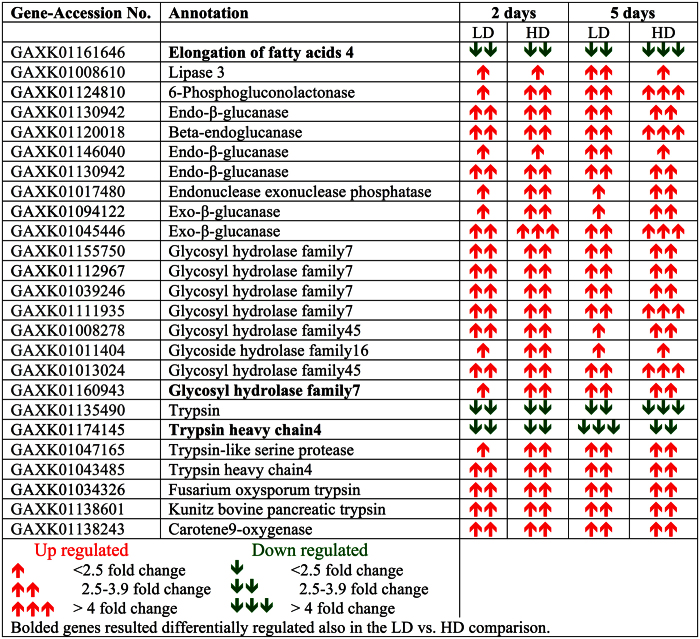
DEGs shared across all experimental conditions. 25 transcripts expressed in *C. finmarchicus* females feeding on *A. fundyense* LD and HD treatments at both 2 and 5 days compared with adult females feeding on the control diet. For each gene, Accession No. (NCBI), *blastx* annotation and relative fold change in expression are listed. The direction of expression (up- down-regulated) and the magnitude are indicated by arrows red = up- and green = down-regulated genes).

A total of 25 DEGs were regulated in *Calanus finmarchicus* females in both LD and HD treatments and at both 2 and 5 days (Fig. 6). Interestingly, 24 of these DEGs (96%) were associated with digestion and included endoglucanases, glycosyl hydrolases, trypsins, a lipase, phosphogluconolactonase and ß-carotene 9 oxygenase (Fig. 6). Most of these DEGs were up-regulated in all experimental conditions with the exception of 2 trypsins that were consistently down-regulated (Fig. 6). Relative expression of these DEGs was similar across treatments and time points with an average 3-fold change in expression compared with the control diet (Fig. 6). The last shared DEG was a member of the elongase family, which is involved in lipid biosynthesis. This elongase (ELOV4) was consistently down-regulated across time points and treatments, with a 6-fold change at 5 days in the HD treatment. A significant dose response was confirmed as this gene was among the DEGs in the LD vs HD comparisons at both 2 and 5 days (Fig. 6).

## Discussion

In pelagic marine ecosystems, nutritional resource limitation due to food quantity or quality can affect copepod populations^1^,^22^,^23^. Sub-optimal food conditions may not increase mortality rates, but might change an individual’s longevity and reproductive potential^24^. In the Gulf of Maine and Bay of Fundy, the calanoid *C. finmarchicus* co-occurs with the saxitoxin-producing dinoflagellate *A. fundyense*, which during bloom conditions can reach densities of 10–100 cells/mL^−1 ^8,9. Since the dinoflagellate does not affect the copepod’s survival, these blooms have not usually been considered an environmental stressor for *C. finmarchicus*^11^. However, more recently it has been shown that *A. fundyense* decreases the reproductive potential of *C. finmarchicus* females^12^. The current study provides further evidence that introduction of *A. fundyense* into the diet of the copepod elicits a cellular stress response. Stress-defense systems are activated in all eukaryotes with the aim to prevent physiological damage caused by a stressor, and the CSR is characterized by large-scale transcriptional changes^17^,^25^,^26^.

Consistent with the study on female reproductive success significant effects were observed at both algal doses, with many shared DEGs between treatments. Although dinoflagellate densities in the HD treatment are extremely rare, the LD treatment is comparable to what *C. finmarchicus* might experience in the Gulf of Maine during high-density blooms^9^,^10^. Our transcriptional response was similar in magnitude to gene expression changes reported in response to natural stressors such as environmental fluctuations in temperature experienced by the killifish, *Austrofundulus limnaeus*^14^. Furthermore, the 2- to 4-fold change in expression reported here in both LD and HD treatments is comparable to responses in the cladoceran *Daphnia* spp. exposed to chemical stressors that did not affect survival (e.g. 1/10 LC50)^27^,^28^.

The time-dependent response observed in the *C. finmarchicus* females can be explained by the initial cellular stress response (CSR) being followed by a cellular homeostatic response (CHR). Physiological adaptation to new environmental conditions varies depending on the specific environmental challenge, and is characterized by fewer differentially expressed genes (DEGs)^16^. After 5 days on the experimental diet, there were many fewer up-regulated DEGs, and few DEGs were shared between time points.

The *A. fundyense* used in this study produced STXs during the experimental period and average daily toxin ingestion rates were 0.3 and 2 ng STX equivalents day^−1^ in the LD and HD treatments, respectively^12^. Surprisingly, detoxification was not a large part of the copepod transcriptional response with only four detoxification DEGs up-regulated at 2 days, and only two at 5 days. In contrast, when insects are exposed to toxic chemicals, a more generalized detoxification response is observed^29^. In *Drosophila melanogaster*, 10% of glutathione S-transferases (GSTs) and cytochrome P450s were differentially expressed in response to short-term exposure (4 h) to natural plant compounds (e.g. caffeine and barbiturate drug phenobarbital)^29^. These detoxification genes were all up-regulated with 2–30 fold change in relative expression compared with the control^29^. The *C. finmarchicus* response is more similar to the response observed in *Daphnia pulex* feeding on the neurotoxin-producing cyanobacterium *Microcystis arguinosa*^30^. Using a microarray with ca. 30,000 genes, the authors reported a large-scale transcriptional response (>2,000 DEGs). However, only six GSTs were up-regulated and all with a modest 2-fold difference in expression compared with the control^30^.

The transcriptional response of *D. pulex* feeding on the cyanobacterium was characterized by many DEGs involved in energy-related processes (e.g. carbohydrate metabolism, protein and ribosome regulation), leading the authors to suggest that this high energetic expenditure might be explained by a reduction in the food assimilation^30^. The cyanobacterium, *M. arguinosa* could be interfering with the cladoceran digestive system, as suggested by the down-regulation of digestive enzymes, e.g., many trypsins^30^. In *C. finmarchicus*, the DEGs that were consistently regulated under all conditions are involved in digestion, with the down-regulation of several trypsins, and the up-regulation of other digestive enzymes. Thus, similar to *D. pulex*, food assimilation in *C. finmarchicus* may be affected by the presence of *A. fundyense* in the diet. In an earlier study, it was suggested that food assimilation might explain a decrease in fecundity in another species of copepod (*Acartia clausi*) feeding on *Alexandrium* spp^31^.

*Calanus finmarchicus* females on the experimental diets seem to have less energy available than the control females as suggested by gene expression differences at 5 days. The cellular homeostatic response was characterized by the down-regulation of transcripts involved in lipid biosynthesis, growth and reproduction and the up-regulation of genes involved in carbohydrate catabolism. Given that females on the control and experimental diets had similar carbon ingestion rates (24 μg C female^−1^ d^−1^)^12^, differences in assimilation might explain the lower energy balance in the experimental females. Thus, if the dinoflagellate compromises food assimilation, *C. finmarchicus* females on the experimental diets would have less energy available, which could have contributed to lower egg production and egg quality as reported by Roncalli *et al*.^12^.

Although *C. finmarchicus* is highly tolerant of *A. fundyense* (i.e. no effect on survival), the dinoflagellate’s effects on the copepod’s physiology and reproductive output could have repercussions on population growth during bloom conditions. The current study was focused on adult females, however, if other developmental stages are affected in a similar way, i.e., down-regulation of lipid biosynthesis pathways, then blooms of *A. fundyense* could interfere with the seasonal accumulation of lipid stores in pre-adults during the summer. Thus, an increase in harmful algal blooms of *A. fundyense* in the Gulf of Maine would be expected to have a negative impact on *C. finmarchicus* populations through a decrease in reproduction^12^ and lower energy balance.

## Methods

### Field collection and maintenance of *Calanus finmarchicus*

*Calanus finmarchicus* were collected using a vertical net tow (75 cm diameter, 560 μm mesh) in July of 2012 in the Gulf of Maine near Mount Desert Rock (Lat: 44° 2′N; Long: 68°3′W) as previously described^12^. Adult females and adult males were transferred into 3.5 L jars at 5–10 individuals per liter with *Rhodomonas* sp. added *ad libitum,* and allowed to acclimate overnight at 10 °C on a 14:10 h light: dark cycle in an incubator (Percival Model I-36VL, Percival Scientific, Inc., Perry, IA, USA).

### Experimental design

To examine the transcriptional response of *Calanus finmarchicus* to *Alexandrium fundyense*, adult females were exposed to three treatments: control, low dose and high dose. In the control group, *C. finmarchicus* were fed the non-toxic flagellate *Rhodomonas* sp. (8000 cells mL^−1^d^−1^). In the “low dose” group (LD) copepods were fed 50 cells mL^−1^d^−1^ *A. fundyense* and 6000 cells mL^−1^d^−1^ *Rhodomonas* sp., which corresponded to a 25:75 proportion by algal volume. The “high dose” (HD) group was fed with 100% of *A. fundyense* at a concentration of 200 cells mL^−1^d^−1^. The three experimental food suspensions had similar carbon content ranging between 304 and 358 μgC L^−1^; albeit slightly higher in the HD treatment (358 μgC L^−1^), non-significant differences were found between the diets^12^.

Adult females were transferred into one of 18 1.5-L containers to be harvested either at 2 days or 5 days. Three biological replicates were set up per treatment (control, low dose and high dose) x sampling day (2 and 5 days) with each containing 15 females. The containers were kept in a Percival Model I-36VL Incubator System (Percival Scientific, Inc., Perry, IA, USA) at 10 °C on a 14:10 h light-dark cycle. At 2 and 5 days, 10 adult females were harvested from each treatment and biological replicate, immediately transferred in 0.5 mL RNAlater (Ambion) and stored at −20 °C until RNA extraction.

### RNA extraction, gene library preparation and sequencing

Total RNA was extracted from the adult females using QIAGEN RNeasy Mini Kit (QIAGEN Inc., Valencia, CA, USA), in conjunction with a Qiashredder column (QIAGEN Inc.), following the instructions of the manufacturer, and with a final elution volume of 30 μl. RNA concentration and quality were checked using an Agilent Model 2100 Bioanalyzer (Agilent Technologies, Inc., Santa Clara, CA, USA). All RNA samples (3 biological replicates × 3 treatments × 2 time points) were of high quality and shipped on dry ice to the University of Georgia Genomics Facility for library preparation and Illumina sequencing. There, the TruSeq RNA sample preparation kit (Illumina) was used to prepare double-stranded multiplexed cDNA libraries starting from the total RNA following manufacturer’s instructions. Briefly, RNA samples were first purified with two oligo-dT selection (poly (A) enrichment using oligo-dT beds) to select for mRNAs, and then fragmented and reverse transcribed into double-stranded complementary cDNA. cDNA libraries were prepared with a 350 bp insert and primed using random hexamers. Each sample was tagged with an indexed adapter prior to shipping to University of Missouri DNA Core Facility ( http://biotech.missouri.edu/dnacore) for sequencing. At the Missouri facility the samples were loaded into a single lane to be run on Illumina HiSeq 2000 instrument using paired-end sequencing (100 bp).

### Mapping of short reads and identification of differentially expressed genes (DEGs)

The 18 RNA-Seq libraries were quality filtered (FASTX Toolkit, version 0.013; http://hannonlab.cshl.edu/fastx_toolkit/) by trimming the first nine and the last 29 bases, and followed by the elimination of low quality reads (cutoff “Phred” score = 20) as well as Illumina adapters. This resulted in the removal of an average of 34% of reads, leaving from 10 to 24 million reads per sample for relative gene expression analysis. Each quality filtered RNA-Seq library was then mapped to a *C. finmarchicus* reference transcriptome (96,090 contigs)^18^ using Bowtie software (version, 2.0.6)^32^ with a 2-nucleotide mismatch tolerance. The reference transcriptome was generated through the *de novo* assembly of over 400 million reads from six developmental stages as described in Lenz *et al*.^18^.

Differential gene expression analysis and calculation of fold-change difference in expression were performed using the BioConductor package edgeR^33^. Transcriptional expression profiles were analyzed for two factors: “time” (2 and 5 days) and “dose” (LD and HD). Prior the statistical analysis, libraries were normalized as implemented by edgeR using the Trimmed mean of M values (TMM) and genes with low expression (<1 count per million) were removed. At each time point, the number of significant differentially expressed genes was established by pairwise comparison of libraries: control vs. LD, control vs. HD and LD vs. HD. For the statistical analysis relative expression was quantified as a ratio of Log_2_ (experimental/control). Genes were recognized as differentially expressed (DEG) using the “exact test” (p < 0.05) and a multiple comparison correction using the Benjamini-Hochberg method (false discovery rate <5%) implemented by edgeR^33^. In the results, the magnitude of differential expression is presented as the fold-change difference between the experimental and the control and between the two experimental diets. The significant differences in the number of up- and down-regulated DEGs between the control vs. LD and HD, and LD vs. HD at 2- and 5-days were determined by using a *X*^2^ test that compared the observed and expected frequencies of number of DEGs per fold-change category. The statistical tests were performed using Prism GraphPad software (v. 6.0).

### Functional annotation

Functional annotations for genes identified as differentially expressed (DEGs) in the control vs. LD, control vs. HD and LD vs. HD comparisons were obtained directly from the annotated *C. finmarchicus* reference transcriptome using blast and gene ontology (GO) analysis^18^. Briefly, using BLAST2GO pipeline (version 2.6.4), annotations for the reference transciptome were obtained against NCBI non-redundant (nr) database (*blastx* algorithm) with maximum E-value of 10^−3 ^18. Gene ontology (GO) annotations for biological and molecular processes and cellular component were assigned using BLAST2GO, here with an E-value of 10^−6^ required for annotation. Enrichment analysis was performed for up- and down-regulated genes with GO terms (293) against the 10,344 genes with assigned GO terms in the *C. finmarchicus* reference transcriptome^18^. The analysis was implemented using the software BLAST2GO (version 2.6.4) performing the Fisher’s Exact Test followed by Multiple Testing correction of False Discovery rate (FDR <5%)^34^. It is important to note that in many cases multiple functions (GO terms) are assigned to individual genes.

### Data availability

Sequence data have been submitted to the National Center of Biotechnology Information (NCBI; www.ncbi.nlm.nih.gov) under the Bioproject PRJNA312028. The list of differentially expressed genes with their annotation (blast) and fold change has been submitted to the Dryad Digital Repository (Provisional DOI: doi:10.5061/dryad.11978) as well as the complete annotation of the C. finmarchicus transcriptome used here as the reference for Bowtie mapping. Additional metadata have been submitted to Biological and Chemical Oceanographic Data Management Office Center of Biotechnology Information (BCO-DMO; www.bco-dmo.org) under the Project CFINTRANSCRIPT (www.bco-dmo.org/dataset/528312).

## Additional Information

**How to cite this article**: Roncalli, V. *et al*. Transcriptomic responses of the calanoid copepod *Calanus finmarchicus* to the saxitoxin producing dinoflagellate *Alexandrium fundyense. Sci. Rep.* **6**, 25708; doi: 10.1038/srep25708 (2016).

## Supplementary Material

Supplementary Information

## Figures and Tables

**Table 1:** Experimental treatments: daily food added to *C. finmarchicus* adult females incubated in 1.5-L containers and harvested at one of two time points (2 or 5 days) for RNA-Seq.

	Treatment		
	Control*Rhodomonas* sp.	Low dose (LD) *Rhodomonas*sp. *Alexandrium fundyense*	High dose (HD)*Alexandrium fundyense*
Target % by amountof carbon*	100% Rho	75% Rho, 25% Alex	100% Alex
Cells mL^−1^	8000	Rho = 6000	200
		Alex = 50	
		**Total** = **6050**	
μg C L^−1^*	304	Rho = 228	358
		Alex = 89	
		**Total** = **317**	
Average daily toxiningestion (ng STXequivalents d^−1^)**	0	0.3	2

*Cellular carbon content for *Rhodomonas* sp. and *Alexandrium fundyense* were computed applying volume to carbon conversions^10^.

**A. fundyense toxicity in stock cultures was measured daily during the duration of the experiment, and toxin ingestion was calculated from grazing rate experiments^10^, which were conducted in parallel to the incubations for the RNA-Seq experiment.

**Table 2:** Summary of differentially expressed genes (DEGs) in *C. finmarchicus* adult females feeding on *A. fundyense* diets (LD, HD) for either 2 or 5 days.

Comparison	2 days	5 days
control vs LD	1,066	567
control vs HD	1,388	962
LD vs HD	470	622

Genes were identified as differently expressed using the “exact” test (p < 0.05) and a multiple comparison correction using Benjamini-Hochberg method (false discovery rate <5%) as implemented by edgeR.

**Table 3:** Gene Ontology (GO) enrichment analysis of up-and down-regulated genes in *C. finmarchicus* feeding on low (LD) and high dose (HD) treatments of *A. fundyense* for 2 days.

Time/treatment	Term description	GO	Category	FDR
**2 days LD**				
	**Up regulated**			
	RNA biosynthetic process	GO:0032774	BP	5.16E-03
	Cellular macromolecule biosynthetic process	GO:0034645	BP	3.01E-03
	Cellular protein metabolic process	GO:0044267	BP	4.73E-02
	Macromolecular complex	GO:0032991	CC	3.01E-02
	Heterocyclic compound binding	GO:1901363	MF	3.01E-02
	Organic cyclic compound binding	GO:0097159	MF	3.01E-02
	Peptidase activity	GO:0008233	MF	3.93E-02
	Transferase activity	GO:0016740	MF	1.10E-02
	Cytoplasmic part	GO:0044444	CC	2.46E-02
	Intracellular membrane-bounded organelle	GO:0043231	CC	3.42E-03
**2 days HD**				
	**Up regulated**			
	Lipid metabolic process	GO:0006629	BP	4.62E-02
	RNA biosynthetic process	GO:0032774	BP	1.58E-03
	Translation	GO:0006412	BP	4.62E-02
	Macromolecular complex	GO:0032991	CC	2.25E-02
	Nucleic acid binding	GO:0003676	MF	1.91E-03
	Nucleotide binding	GO:0000166	MF	3.79E-02
	Signal transduction	GO:0007165	BP	1.55E-02
	Cytosol	GO:0005829	CC	2.91E-02
	Extracellular region	GO:0005576	CC	5.75E-03
	Nucleus	GO:0005634	CC	1.23E-03
	**Down regulated**			
	Cellular metabolic process	GO:0044237	BP	2.48E-02
	Single-organism cellular process	GO:0044763	BP	2.88E-02

Gene ontology term (GO); Category: Biological process (BP), Molecular function (MF) and Cellular component (CC); false discovery rate (FDR) after Fisher exact test using FDR <5% as cutoff.

**Table 4:** Gene Ontology (GO) enrichment analysis of down-regulated genes in *C. finmarchicus* feeding on low (LD) and high (HD) dose treatments of *A. fundyense* for 5 days.

Time/treatment	Term description	GO term	Category	FDR
**5 days LD**				
	**Down regulated**			
	Lipid metabolic process	GO:0006629	BP	9.72E-03
	Protein metabolic process	GO:0019538	BP	4.52E-02
	Cell communication	GO:0007154	BP	4.52E-02
	Single organism signaling	GO:0044700	BP	3.92E-02
	Nucleobase-containing compound metabolic process	GO:0006139	BP	3.92E-02
**5 days HD**				
	**Down regulated**			
	Lipid metabolic process	GO:0006629	BP	4.62E-02
	RNA biosynthetic process	GO:0032774	BP	1.58E-03
	Translation	GO:0006412	BP	4.62E-02
	Macromolecular complex	GO:0032991	CC	2.25E-02
	Nucleic acid binding	GO:0003676	MF	1.91E-03
	Nucleotide binding	GO:0000166	MF	3.79E-02
	Signal transduction	GO:0007165	BP	1.55E-02
	Reproduction	GO:0000003	BP	3.84E-02
	Cytosol	GO:0005829	CC	2.91E-02
	Extracellular region	GO:0005576	CC	5.75E-03
	Nucleus	GO:0005634	CC	1.23E-03

Gene ontology term (GO); Category: Biological process (BP), Molecular function (MF) and Cellular component (CC); false discovery rate (FDR) after Fisher exact test using FDR <5% as cutoff.
