# Supplementary material for: Transcriptomic responses of the calanoid copepod Calanus finmarchicus to the saxitoxin producing dinoflagellate Alexandrium fundyense
Source: Sci Rep. 2016 May 16;6:25708. doi: 10.1038/srep25708 (PMC4867593; doi:10.1038/srep25708)
Supplement: Supplementary Information [file srep25708-s1.doc]

| Samples | Raw reads (#) | Discarded reads (%) | Reads for mapping (#) | Overall alignment rate (#) | Total mapped reads (#) | Mapped reads 1 time (#) | Mapped reads 1 time (%) | Mapped reads > 1time (#) | Mapped reads > 1 time (%) |
| --- | --- | --- | --- | --- | --- | --- | --- | --- | --- |
| **2 days** |  |  |  |  |  |  |  |  |  |
| C r1 | 22,678,446 | 33 | 15,256,487 | 71.4 | 10,898,526 | 10,799,129 | 70.8 | 99,397 | 0.6 |
| C r2 | 15,334,636 | 34 | 10,098,479 | 71.1 | 7,176,817 | 7,110,892 | 70.4 | 65,925 | 0.6 |
| C r3 | 20,164,880 | 33 | 13,447,296 | 70.7 | 9,511,875 | 9,419,018 | 70.7 | 92,857 | 0.7 |
| LD r1 | 24,397,138 | 35 | 15,852,474 | 71.6 | 11,350,347 | 11,246,340 | 70.9 | 104,007 | 0.7 |
| LD r2 | 32,032,448 | 35 | 20,922,762 | 70.9 | 14,836,541 | 14,673,615 | 70.1 | 162,926 | 0.8 |
| LD r3 | 26,732,596 | 34 | 17,615,813 | 71.7 | 12,635,344 | 12,513,350 | 71 | 121,994 | 0.7 |
| HD r1 | 25,371,346 | 35 | 16,564,095 | 71.5 | 11,840,963 | 11,735,536 | 70.8 | 105,427 | 0.6 |
| HD r2 | 36,624,954 | 35 | 23,924,809 | 72.5 | 17,335,549 | 17,178,945 | 71.8 | 156,604 | 0.6 |
| HD r3 | 22,361,678 | 36 | 14,346,106 | 71.6 | 10,274,526 | 10,171,571 | 70.9 | 102,955 | 0.7 |
| **5 days** |  |  |  |  |  |  |  |  |  |
| C r1 | 24,950,224 | 36 | 15,972,067 | 71.4 | 11,407,748 | 11,307,484 | 70.8 | 100,264 | 0.6 |
| C r2 | 33,605,768 | 35 | 21,727,284 | 71.5 | 15,536,963 | 15,404,299 | 70.9 | 132,664 | 0.6 |
| C r3 | 36,757,738 | 34 | 24,354,372 | 71.6 | 17,447,497 | 17,294,204 | 71 | 153,293 | 0.6 |
| LD r1 | 35,824,224 | 35 | 23,325,237 | 71.1 | 16,583,915 | 16,443,476 | 70.5 | 140,439 | 0.6 |
| LD r2 | 22,073,822 | 34 | 14,518,531 | 71.7 | 10,411,305 | 10,311,783 | 71 | 99,522 | 0.7 |
| LD r3 | 30,768,506 | 35 | 20,111,901 | 71.3 | 14,348,892 | 14,229,233 | 70.7 | 119,659 | 0.6 |
| HD r1 | 26,147,888 | 34 | 17,172,169 | 71.4 | 12,257,785 | 12,143,290 | 70.7 | 114,495 | 0.7 |
| HD r2 | 22,435,778 | 32 | 15,283,042 | 71.8 | 10,952,138 | 10,851,978 | 71 | 100,160 | 0.7 |
| HD r3 | 26,186,738 | 35 | 17,037,038 | 71 | 12,095,361 | 11,985,806 | 70.3 | 109,555 | 0.6 |
| * % of reads mapping | | | | | | | | | |

**Transcriptomic responses of the calanoid copepod *Calanus finmarchicus* to the saxitoxin producing dinoflagellate *Alexandrium fundyense***

Vittoria Roncalli, Matthew C. Cieslak and Petra H. Lenz

**Supplementary Table 1.** Summary of RNA-sequencing and mapping results for *Calanus finmarchicus* adult females feeding on *Rhodomonas* sp. (C) and *A. fundyense* (LD, HD) diets for 2 and 5 days. Raw reads were quality filtered and trimmed prior to mapping to a *C. finmarchicus* reference transcriptome^16^ using Bowtie software. C=control diet, LD= Low dose diet, HD= High dose diet. For each treatment 3 biological replicates were collected (r1, r2, r3).
